# Rational design of alcoholic fermentation targeting extracellular carbon

**DOI:** 10.1038/s41538-023-00215-0

**Published:** 2023-07-21

**Authors:** Daisuke Watanabe, Mikiya Kawashima, Naoya Yoshioka, Yukiko Sugimoto, Hiroshi Takagi

**Affiliations:** Division of Biological Science, Graduate School of Science and Technology, Nara Institute of Science and Technology, Ikoma, Nara Japan

**Keywords:** Applied microbiology, Metabolic engineering, Fungal genetics

## Abstract

Breeding yeast strains for industrial alcoholic fermentation requires laborious screening due to the lack of in vivo modification strategies. Here we show that quiescence-specific cell wall thickening via synthesis of a major component, 1,3-β-glucan, critically antagonizes cellular fermentation ability by sequestering the available cytoplasmic carbon sources. This study provides insights into glycolytic control and reports an effective and reliable rational fermentation design.

The current understanding of glycolysis comes from the study of yeast alcoholic fermentation^1^. Glycolytic control is a central issue in cellular metabolism^2^. The robustly optimized glycolytic behavior of yeast, known as the Crabtree effect^3^, hinders the development of breeding technology for altering the alcoholic fermentation capacity. Protein phosphatase 2A (PP2A) complexed with the B55δ-type regulatory subunit (Cdc55p) is solely responsible for the outstanding glycolytic activity of *Saccharomyces cerevisiae* sake yeast strains^4^. High glucose levels activate PP2A^B55δ^ in both yeasts and mammals. In humans, hepatic glucose metabolism triggers PP2A^B55δ^-dependent dephosphorylation of phosphofructokinase-2/fructose-2,6-bisphosphatase, promoting the production of the key glycolytic activator, fructose-2,6-bisphosphate^5,6^. In *S. cerevisiae*, however, its role in the glycolytic control remains questionable^7^. Therefore, the mechanism underlying PP2A^B55δ^-mediated yeast alcoholic fermentation remains unknown.

RNA-seq of *S. cerevisiae cdc55*Δ cells, specifically defective in the PP2A^B55δ^ function, on entry into cellular quiescence at the initial fermentation stage revealed upregulation of stress-responsive genes under the control of redundant transcription factors, such as Msn2p, Msn4p (Msn2/4p), and Gis1p, functionally associated with mammalian FoxO^8^ (Supplementary Fig. 1, Supplementary Tables 1 and 2). Loss of Cdc55p led to a significant increase in the expression of these genes in an Msn2/4p-dependent manner (Fig. 1a, b, Supplementary Fig. 2). This was unexpected because loss of Cdc55p reduces the acute stress-responsive expression of identical genes in exponentially proliferating cells (Supplementary Fig. 3)^9^. Therefore, PP2A^B55δ^ exhibits opposing effects in Msn2/4p activation in the quiescence and proliferation phases (Supplementary Fig. 4). Deletion of *MSN2/4* genes in the *cdc55*Δ background almost fully recovered the poor fermentation phenotype (Fig. 1c and Supplementary Fig. 5), suggesting that Msn2/4p is a major glycolytic inhibitor.

**Fig. 1:**
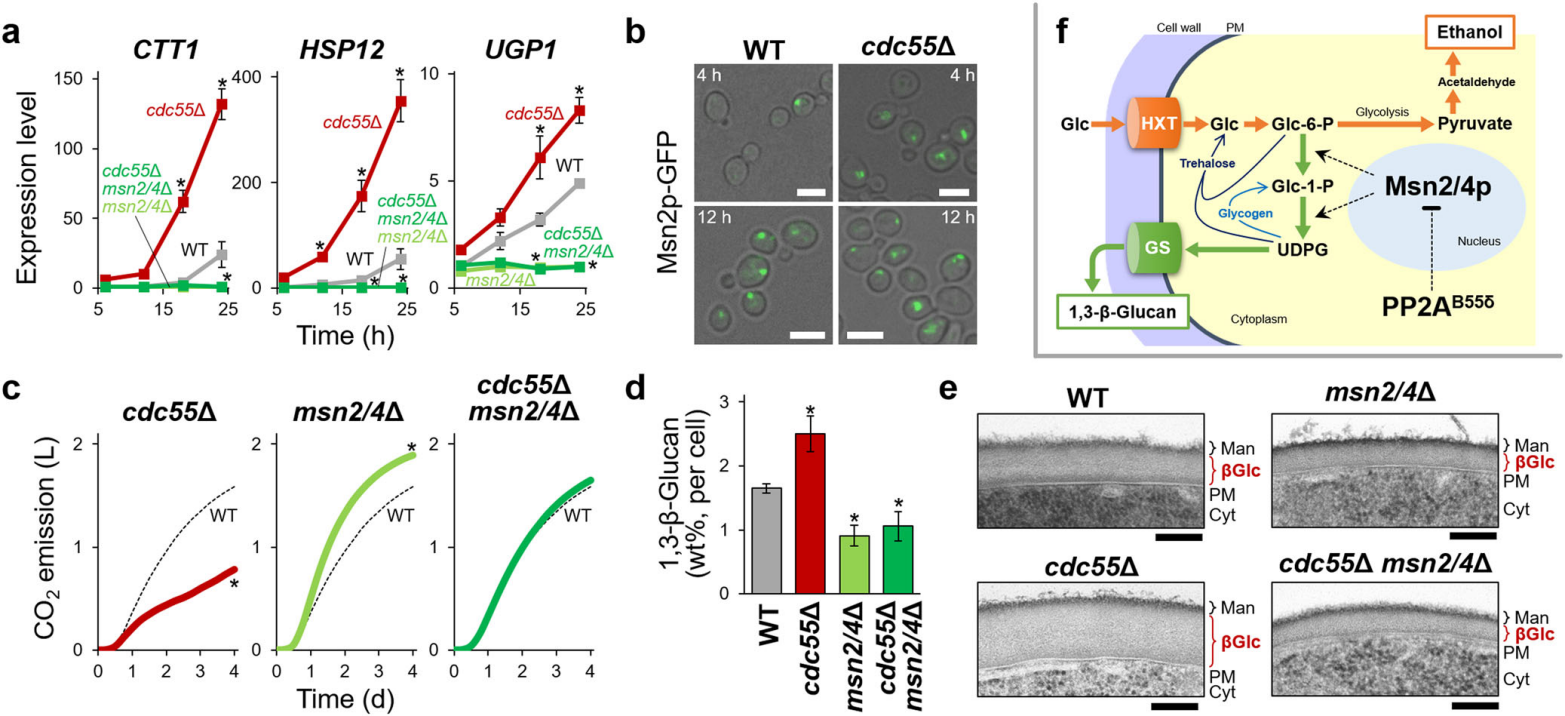
Yeast PP2A^B55δ^ positively regulates alcoholic fermentation via inhibiting Msn2/4p-dependent cell wall thickening. **a** Loss of PP2A^B55δ^ (*cdc55*Δ) enhances the expression of Msn2/4p-targeted genes at the early stage of fermentation. The y axis stood for the relative expression levels in comparison to the wild-type 6 h samples. The values are the means and standard deviations of three independent experiments. **b** *cdc55*Δ　enhances nuclear localization of Msn2p-GFP at the early stage of fermentation. Bars, 5 μm. **c** The low fermentation capacity of *cdc55*Δ cells is mostly dependent on Msn2/4p. WT, wild type. (**d, e**) *cdc55*Δ enhances Msn2/4p-dependent 1,3-β-glucan synthesis. **d** Cellular 1,3-β-glucan levels at 24 h after inoculation. The values are the means and standard deviations of three independent experiments. **e** TEM observation of yeast cells at 24 h after inoculation. Man, mannoproteins; βGlc, β-glucan layer; PM, plasma membrane; Cyt, cytoplasm. Bars, 100 nm. **f** Hypothetical model of extracellular carbon sequestering via PP2A^B55δ^ and Msn2/4p. Glc glucose, Glc-6-P glucose-6-phosphate, Glc-1-P glucose-1-phosphate, UDPG UDP-glucose, HXT hexose transporter, GS 1,3-β-glucan synthase. Asterisks denote values that are statistically different from WT, as determined using Student’s *t-test*, *p* < 0.05.

Msn2/4p upregulates genes of the UDP-glucose synthesis pathway, which directly branches from glycolysis via glucose-6-phosphate^10^. UDP-glucose is mostly used as a substrate for the biosynthesis of trehalose, glycogen, and 1,3-β-glucan in *S. cerevisiae*. Cytoplasmic trehalose and glycogen shunts are transiently activated upon acute stress or quiescence entry; therefore, deletion of neither trehalose nor glycogen synthesis genes contributes positively to alcoholic fermentation^11^. In contrast, 1,3-β-glucan accumulates during the fermentation period and its extracellular degradation does not immediately refuel glycolysis. Thus, 1,3-β-glucan synthesis in quiescent cells may be involved in metabolic control through the discharge of fermentable carbon sources into the extracellular space. Cell wall quantification (Fig. 1d) and transmission electron microscopy (Fig. 1e) revealed marked 1,3-β-glucan thickening in fermenting *cdc55*Δ cells and its complete absence in *cdc55*Δ *msn2/4*Δ cells, inversely correlated with the fermentation rates. Therefore, hyperactivated 1,3-β-glucan synthesis is responsible for the poor fermentation phenotype of *cdc55*Δ cells.

The 1,3-β-glucan synthesis pathway in *S. cerevisiae*, defined in proliferative cells, comprises the cell wall sensor, Wsc1p, guanine nucleotide exchange factor Rom2p, and regulatory (Rho1p) and catalytic (Fks1/2p) subunits of 1,3-β-glucan synthase^12^. Quantification of 1,3-β-glucan in fermenting cells revealed a similar pathway; however, the responsible cell wall sensors were homologous Wsc3/4p (Fig. 2a). Expression of the *WSC3/4* genes was significantly induced under the control of Msn2/4p (Supplementary Fig. 6), indicating that Wsc3/4p is a quiescence-specific cell wall sensor (Supplementary Fig. 7). Deletion of *WSC3*, *WSC4*, *ROM2*, *FKS1*, or *FKS2*, but not *wsc1*Δ, significantly increased the fermentation rate (Fig. 2b and Supplementary Fig. 8, 9). Therefore, cell wall 1,3-β-glucan has a pivotal role in carbon/energy storage in *S. cerevisiae*.

**Fig. 2:**
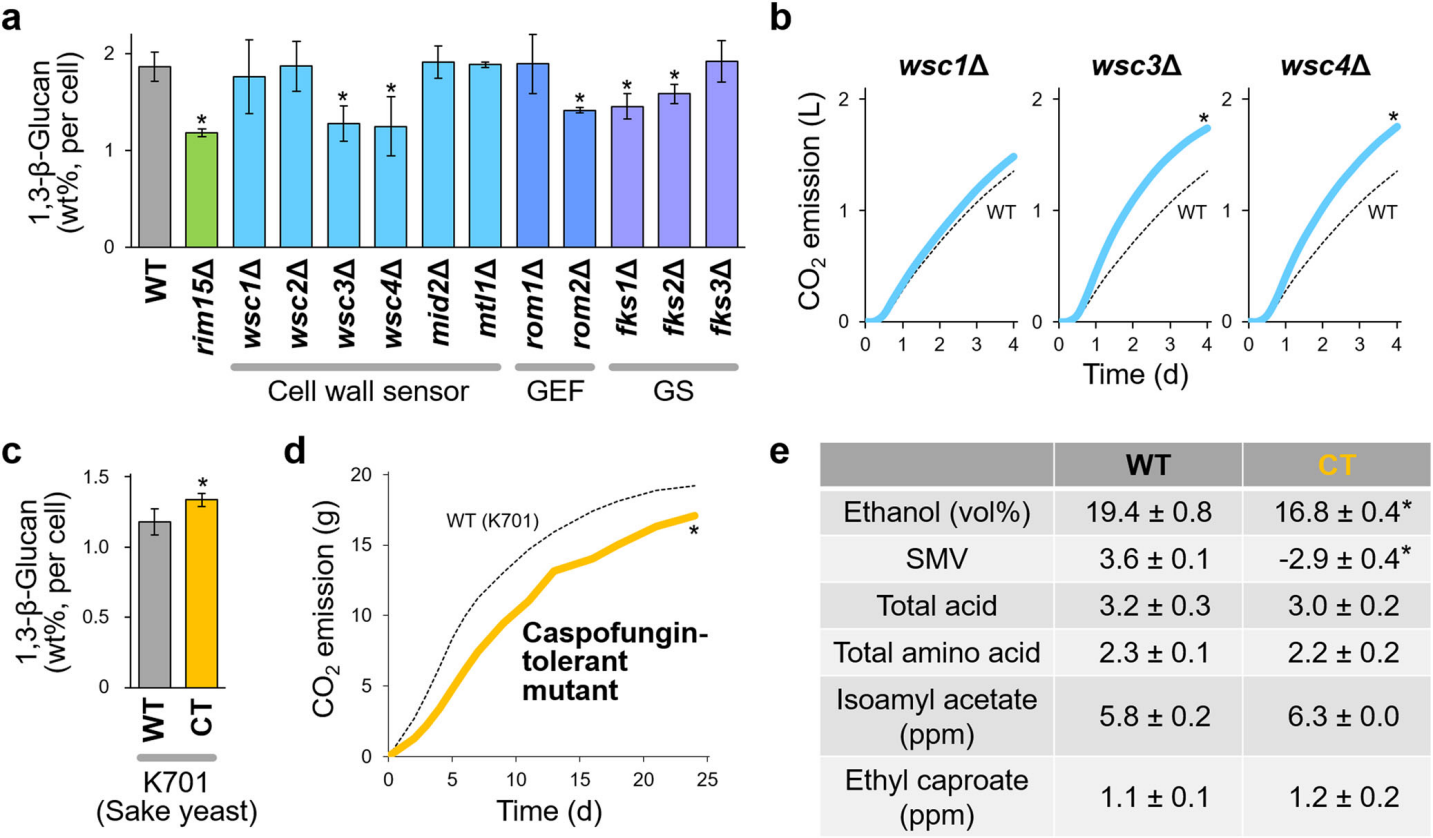
Rational fermentation design targeting 1,3-β-glucan synthesis. **a** Identification of the 1,3-β-glucan synthetic pathway during alcoholic fermentation, based on cellular 1,3-β-glucan levels at 24 h after inoculation. GEF, guanine nucleotide exchange factor for Rho1p; GS, catalytic subunit of 1,3-β-glucan synthase. The values are the means and standard deviations of three or more independent experiments. The deletion of *RHO1*, the sole regulatory subunit of 1,3-β-glucan synthase, was not tested here due to lethality. **b** Improvement of fermentation capacity by deleting the 1,3-β-glucan synthetic pathway genes. WT, wild type. **c**–**e** Non-GM yeast strain designed for low-alcohol sake making. **c** Cellular 1,3-β-glucan levels at 24 h after inoculation. The values are the means and standard deviations of three independent experiments. **d** Fermentation capacity during sake fermentation. **e** Sake parameters after fermentation was completed. Sake meter value (SMV) corresponds to the density of sake; positive and negative values indicate that the density is lower and higher than water, respectively. WT wild type, CT caspofungin-tolerant mutant. Asterisks denote values that are statistically different from WT, as determined using Student’s *t-test*, *p* < 0.05.

To address fermentation design without genetic modification (GM) techniques, ethylmethanesulfonate-mutagenized mutants of the laboratory strain, X2180 and the industrial sake yeast strain, K701, which had acquired a high tolerance against caspofungin, a lipopeptide antifungal 1,3-β-glucan synthase inhibitor^13^, were obtained (Fig. 2c). These caspofungin-tolerant mutants exhibited slower fermentation rates (Fig. 2d, Supplementary Fig. 10, 11) and lower ethanol yields than the parental strains (Fig. 2e). The mutation did not significantly affect other major sake parameters (e.g. total acid and amino acid affecting the taste and ester compounds affecting the aroma) in the sake brewing test. Post-fermentation physical processes, such as vacuum distillation and reverse osmosis, are widely used to industrially reduce ethanol in alcoholic beverages to meet quality, financial, or health demands^14,15^. The fermentation design developed for *S. cerevisiae* successfully altered ethanol content without affecting the overall sensory balance and quality of alcoholic beverages. We also note that a non-GM mutant less tolerant to caspofungin achieved a further higher fermentation rate than K701; this finding could be promising for efficient bioethanol production (Supplementary Fig. 12).

The economic importance of alcoholic fermentation is acceleratingly increasing in terms of not only brewing/winemaking but also industrial production of biofuels and non-fuel ethanol. However, sufficient attention has not been paid to fermentation performance of individual yeast cells so far. Our study identified yeast cell wall as an untouched carbon reservoir to be rationally modified for improving the cost and efficiency of ethanol production. This idea could have important implications for any kind of bioproduction using polysaccharide-enveloped microorganisms, such as fungi, algae, and bacteria^16,17^. Cell wall-targeted carbon metabolic engineering is a challenge for efficient and sustainable biomanufacturing. Our goal is deep understanding the whole map of the regulatory mechanism for alcoholic fermentation in *S. cerevisiae*. Since we expect that various molecular mechanisms are involved, truly “rational design” should be possible by integrating our understanding of these mechanisms. We believe that this study is the key first step toward that end.

## Methods

### Yeast strains

*Saccharomyces cerevisiae* laboratory strain BY4741 and its single-deletion mutants, *wsc1*Δ, *wsc2*Δ, *wsc3*Δ, *wsc4*Δ, *mid2*Δ, *mtl1*Δ, *rom1*Δ, *rom2*Δ, *fks1*Δ, *fks2*Δ, and *fks3*Δ, were obtained from the European *Saccharomyces cerevisiae* Archive for Functional Analysis (Euroscarf), Germany. Disruption of the *MSN2/4* genes in BY4741 was performed using a PCR-based method^18^ with the gene-specific primer pairs, MSN2 + URA3-Fw (5'-GTA TCT TCC TCA TAT TTT TCG GGA AGA TCA CAA CAG TAG CAA GGT ATT TCA TAC GCC AAG AGG CTA CGA TTC GGT AAT CTC CGA G-3')/MSN2 + URA3-Rv (5'-AAC AAT AAG CCG TAA GCT TCA TAA GTC ATT GAA CAG AAT TAT CTT ATG AAG AAA GAT CTA CGA ATT AGT AAT AAC TGA TAT AAT TAA ATT G-3') and Fw Dmsn4-kanMX (5'-TTC GGC TTT TTC TTT TCT TAT TAA AAA CAA TAT AAT GGG TAA GGA AAA GAC TCA-3')/Rv Dmsn4-kanMX (5'-TAG CTT GTC TTG CTT TTA TTT GCT TTT GAC CTT ATT TTT AGA AAA ACT CAT CGA GCA-3') to generate BY4741 *msn2*Δ*::URA3 msn4*Δ*::kanMX* (BY4741 *msn2/4*Δ). The BY4741 *MSN2-GFP::HIS3* strain was kindly provided by Prof. Ted Powers (Department of Molecular and Cellular Biology, University of California, Davis). Disruption of *CDC55* or *RIM15* was performed as previously described^4^. Yeast cells were grown in YPD medium (1% yeast extract, 2% peptone, and 2% glucose) at 30 °C.

Sake yeast strain Kyokai no. 701 (K701) was provided by the Brewing Society of Japan (SBJ). To isolate the caspofungin-tolerant sake yeast strain, K701 was grown in YPD medium at 30 °C upto the stationary phase; 2.5 mL of cell suspension was mixed with 125 μL of 5% ethyl methanesulfonate in phosphate buffer (pH 7.0). After 60 min, 2.6 mL of 10% sodium thiosulfate was added to stop mutagenesis. The cells were collected, washed twice with sterile water, and plated on YPD agar plates containing 5 μg/mL caspofungin. The plates were incubated at 30 °C; a robustly growing colony was isolated as a caspofungin-tolerant mutant strain.

### RNA-seq analysis

BY4741 wild-type and *cdc55*Δ cells were precultured in YPD medium overnight at 30 °C, inoculated into 50 mL of YPD20 medium (1% yeast extract, 2% peptone, and 20% glucose) at a final optical density at a wavelength of 600 nm (OD_600_) of 0.1, and then further incubated at 30 °C without shaking for 6 h. Then, the cells were collected, immediately frozen in liquid nitrogen, and subjected to total RNA extraction and RNA-seq analysis in the Bioengineering Lab. Co., Ltd. (Sagamihara, Japan). Total RNA was extracted using RNeasy Mini Kit (Qiagen) according to the manufacturer’s instructions. The integrity of total RNA was assessed via a 5200 Fragment Analyzer System (Agilent) run using the Agilent HS RNA Kit (Agilent), and only RNAs with RNA integrity numbers (RIN) above 9.5 were used for subsequent treatments and sequencing. After rRNA depletion using riboPOOL (siTOOLs Biotech), circularized DNA libraries were prepared using MGIEasy RNA Directional Library Prep Set (MGI Tech) and MGIEasy Circularization Kit (MGI Tech), and then sequenced using a DNBSEQ-G400 sequencer (MGI Tech). Approximately 30 million pair-end reads (2 × 100 bp) were sequenced for each sample. Raw sequences were demultiplexed using Cutadapt. The sequencing reads were mapped to the *S. cerevisiae* S288C reference genome using hisat2 (ver. 2.2.1). After normalization using iDEGES^19^, differentially expressed genes (DEGs; cutoff *q*-value < 0.05) were identified using the R package edgeR^20^. The gene expression data are shown as reads per kilobase of transcript per million mapped reads (RPKM).

### qRT-PCR analysis

To test gene expression under acute stress, yeast cells were pre-cultured in YPD medium overnight at 30 °C, inoculated into 60 mL of YPD medium at a final OD_600_ of 0.1, and further incubated at 30 °C with vigorous shaking until the OD_600_ reached ~1.0. Half of the cell suspension was treated with high osmotic stress (0.4 M NaCl) for 30 min. To test gene expression under fermentative conditions, yeast cells were precultured in YPD medium overnight at 30 °C, inoculated into YPD20 medium at a final OD_600_ of 0.1, and further incubated at 30 °C without shaking for 24 h. Then, the cells were collected, immediately frozen in liquid nitrogen, and subjected to total RNA extraction using an RNeasy mini kit (Qiagen). cDNA was synthesized using a high-capacity cDNA reverse transcription kit (Applied Biosystems). Each PCR mix (25 μL) contained cDNA (10 ng), 0.1 M primers, and 12.5 μL PCR master mix (2x SYBR Green qPCR master mix; Thermo Fisher Scientific). The primers pairs used are listed in Supplementary Table 3. Gene-specific quantitative real-time PCR (qRT-PCR) was performed on a LightCycler 96 system (Roche Diagnostics). Delta cycle threshold (Δ*C*_*T*_) values were calculated by subtracting the Δ*C*_*T*_ value of the *ACT1* gene from the Δ*C*_*T*_ value for each target gene. Fold-changes were calculated using the 2^-ΔΔ*CT*^ method^21^.

### Fluorescent microscopy

BY4741 wild-type or *cdc55*Δ strain expressing Msn2p-GFP was pre-cultured in YPD medium overnight at 30 °C, inoculated into YPD20 medium at a final OD_600_ of 0.1, and further incubated at 30 °C without shaking for 24 h. Collected cells were immediately observed under a fluorescent microscope Axiovert 200 M (Carl Zeiss) for GFP signal. Images were captured with a HBO 100 Microscope Illuminating System (Carl Zeiss) digital camera.

### Fermentation tests

To determine the fermentation rates in YPD20 medium, yeast cells were precultured in YPD medium at 30 °C overnight, inoculated into 50 mL of YPD20 medium at a final OD_600_ of 0.1, and then further incubated at 30 °C without shaking. Fermentation progression was monitored continuously by measuring the volume of evolved carbon dioxide gas using a Fermograph II apparatus (Atto). To determine fermentation rates during sake making, a single-step sake mash was prepared by mixing 36.4 g pregelatinized rice, 9.6 g dried koji (rice cultivated with *Aspergillus oryzae*), 50 μL of 90% lactic acid, and 87 mL water containing yeast cells at a final OD_600_ of 1.0; this was incubated at 15 °C for 24 d without shaking. Fermentation was monitored continuously by measuring the weight loss of sake mash. Ethanol concentrations and other sake parameters were determined using the standard methods established by the National Tax Agency of Japan (Official Analysis Method of National Tax Agency (in Japanese)).

### Quantification of 1,3-β-glucan

Yeast cell wall 1,3-β-glucan was quantified using a β-glucan assay kit (yeast and mushroom; Megazyme), according to the manufacturer’s protocol. All data were normalized to the wet weight of the cell pellets.

### TEM

Electron microscopy was performed by Tokai Electron Microscopy, Inc. (Nagoya, Japan) based on a rapid freezing and freeze-fixation method. Yeast cell pellets sandwiched between copper disks were quickly frozen in liquid propane at −175 °C and then treated with 2% glutaraldehyde, 1% tannic acid in ethanol, and 2% distilled water at -80 °C for 48 h. The samples were dehydrated using anhydrous ethanol, infiltrated with a 1:1 mixture of propylene oxide and resin (Quetol-651; Nisshin EM Co., Tokyo, Japan) at room temperature for 3 h, then infiltrated with 100% Quetol-651 at room temperature for 3 h, followed by polymerization at 60 °C for 48 h. The polymerized resins were ultra-thin-sectioned at a thickness of 70 nm using an ultramicrotome (Ultracut UCT; Leica). The sections were placed on copper grids, which were subjected to staining with 2% uranyl acetate for 15 min and with lead stain solution (Sigma-Aldrich) for 3 min. The samples were observed using a transmission electron microscope (JEM-1400 Plus; JEOL). Images were acquired using a charge-coupled device (CCD) camera (EM-14830RUBY2; JEOL).

### Statistical analysis

The results are presented as the mean ± standard deviation of three or more independent experiments. Statistical significance was evaluated using Student’s *t*-test. *p* < 0.05 was considered statistically significant.

### Reporting summary

Further information on research design is available in the Nature Research Reporting Summary linked to this article.

## Supplementary information

Supplementary Information

Reporting Summary

## Data Availability

All sequences determined in the RNA-seq analysis of BY4741 wild-type and *cdc55*Δ cells were deposited in the NCBI GEO repository under accession number GSE220072.
