# Supplementary material for: Rational design of alcoholic fermentation targeting extracellular carbon
Source: NPJ Sci Food. 2023 Jul 21;7:37. doi: 10.1038/s41538-023-00215-0 (PMC10361962; doi:10.1038/s41538-023-00215-0)
Supplement: Supplementary file 1 — Supplementary Information [file 41538_2023_215_MOESM1_ESM.pdf]

Supplementary Figure 1 (Watanabe *et al.*)

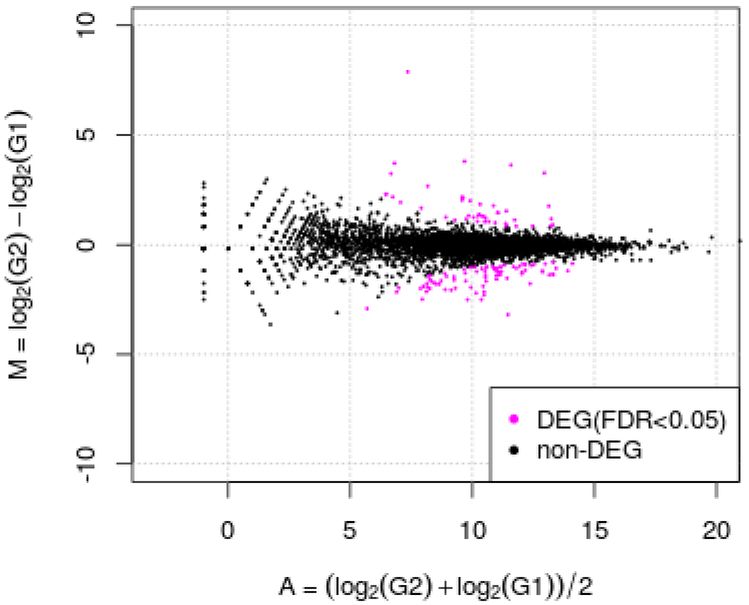

Supplementary Figure 1 Distribution of differentially expressed genes (DEGs) between WT and *cdc55*Δ in the early stage of alcoholic fermentation. The MA plot shows the pairwise comparison between the samples. DEGs (q-value < 0.05) are highlighted in pink. G1 and G2 correspond to the expression levels of WT and *cdc55*Δ samples, respectively.

Supplementary Figure 2 (Watanabe *et al.*)

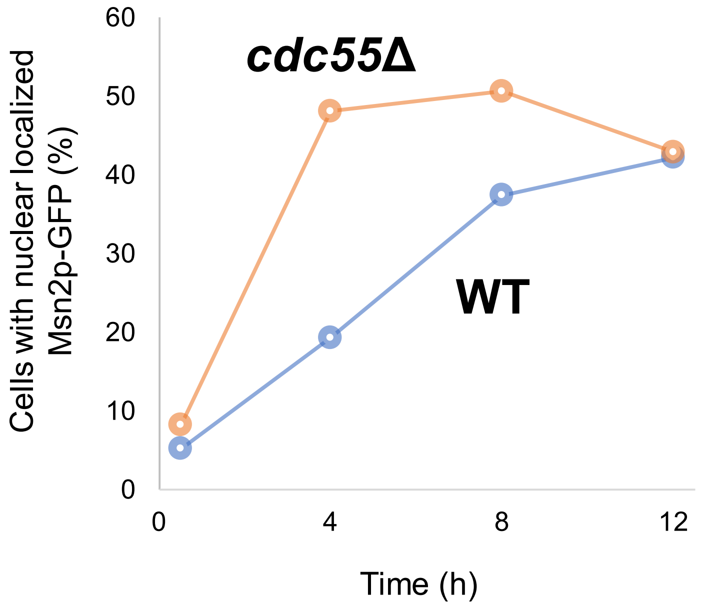

Supplementary Figure 2 *cdc55*Δ enhances nuclear localization of Msn2p-GFP at the early stage of alcoholic fermentation. More than 100 wild-type (blue) or *cdc55*Δ (orange) cells expressing Msn2p-GFP were observed under fluorescent microscope.

Supplementary Figure 3 (Watanabe *et al.*)

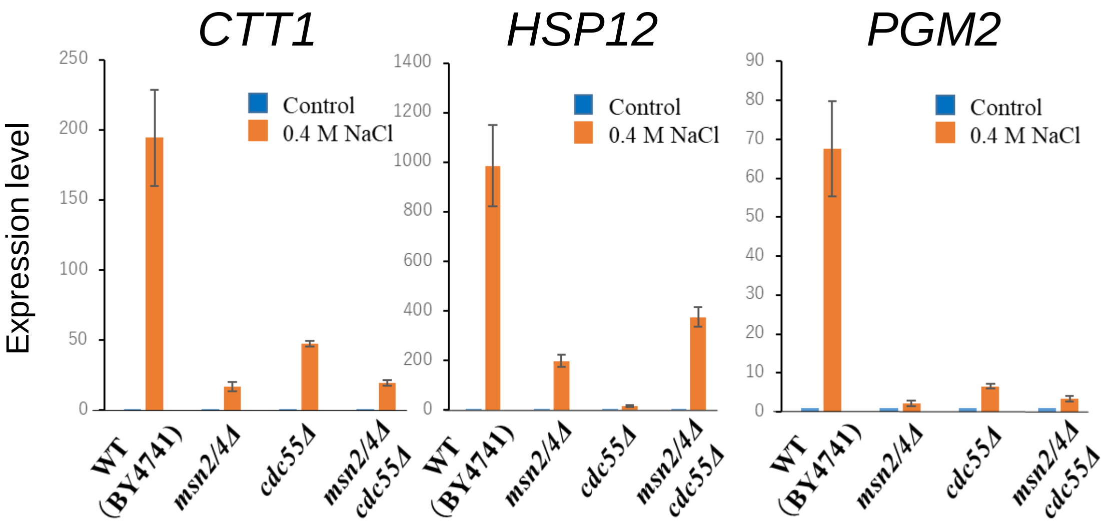

Supplementary Figure 3 *cdc55*Δ decreases expression levels of the Msn2/4p-targeted genes under acute stress. Cells were treated with high osmotic stress and were subjected to qRT-PCR analysis. The expression levels of stressed cells (orange bars) were normalized by those of unstressed cells (blue bars). The values are the means and standard deviations of three independent experiments.

Supplementary Figure 4 (Watanabe *et al.*)

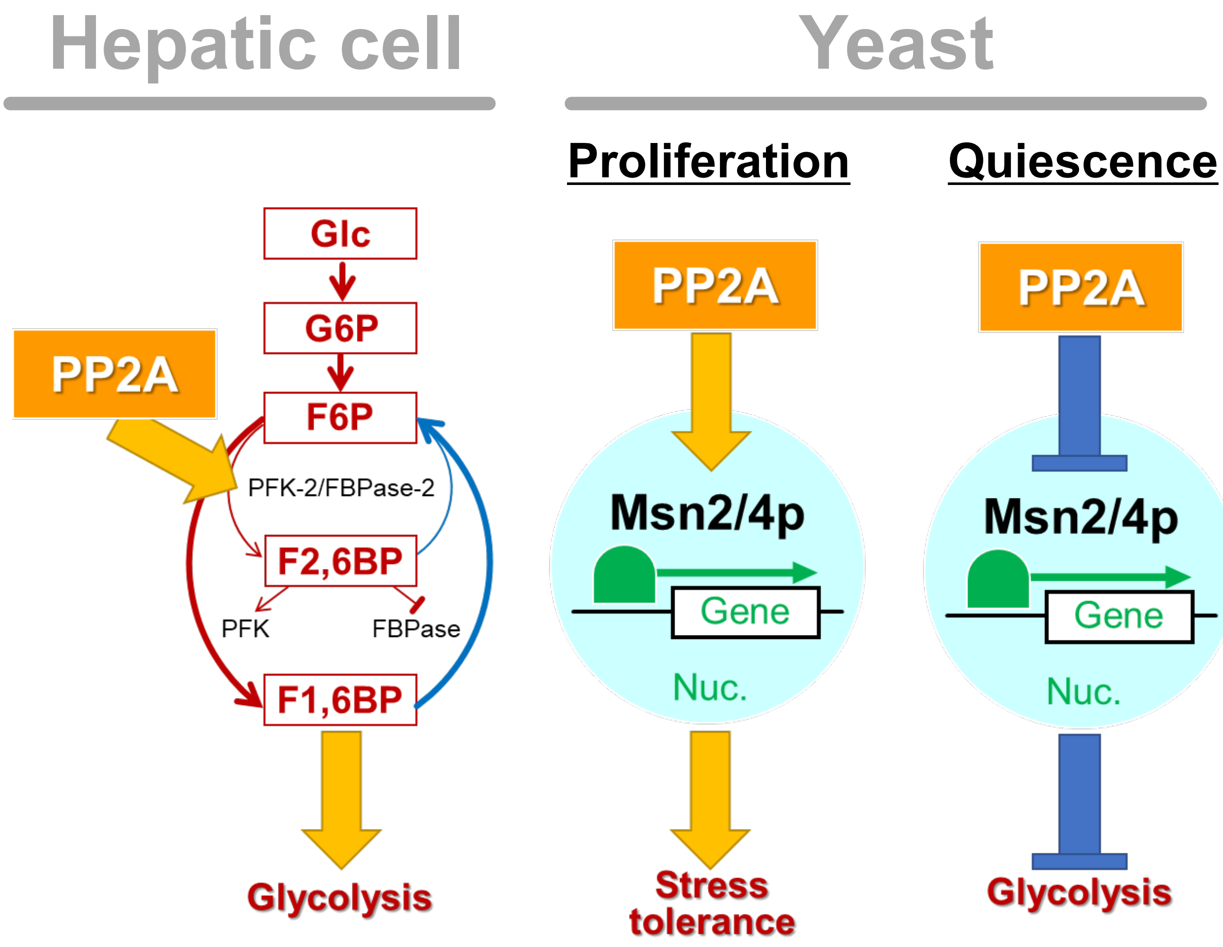

Supplementary Figure 4 Yeast PP2A$^{B55\delta}$'s dual opposite roles toward Msn2/4p between proliferation and quiescence phases. In hepatic cells, PP2A$^{B55\delta}$ activates glycolysis by promoting production of fructose-2,6-bisphosphate. In yeast *S. cerevisiae*, PP2A$^{B55\delta}$ activates glycolysis through inactivating Msn2/4p. This is intriguing because in proliferating yeast cells, PP2A$^{B55\delta}$ activates Msn2/4p in response to acute environmental changes to enhance stress tolerance.

Supplementary Figure 5 (Watanabe *et al.*)

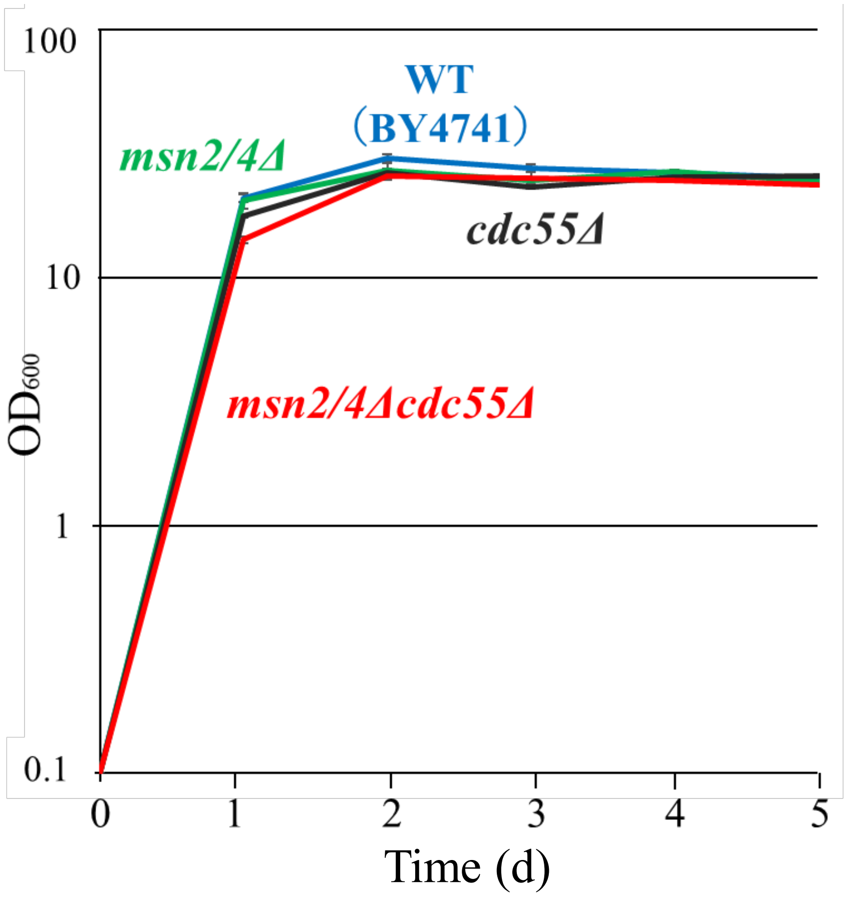

Supplementary Figure 5 Neither *cdc55*Δ nor *msn2/4*Δ does not alter yeast growth during alcoholic fermentation. The values are the means and standard deviations of three independent experiments.

Supplementary Figure 6 (Watanabe *et al.*)

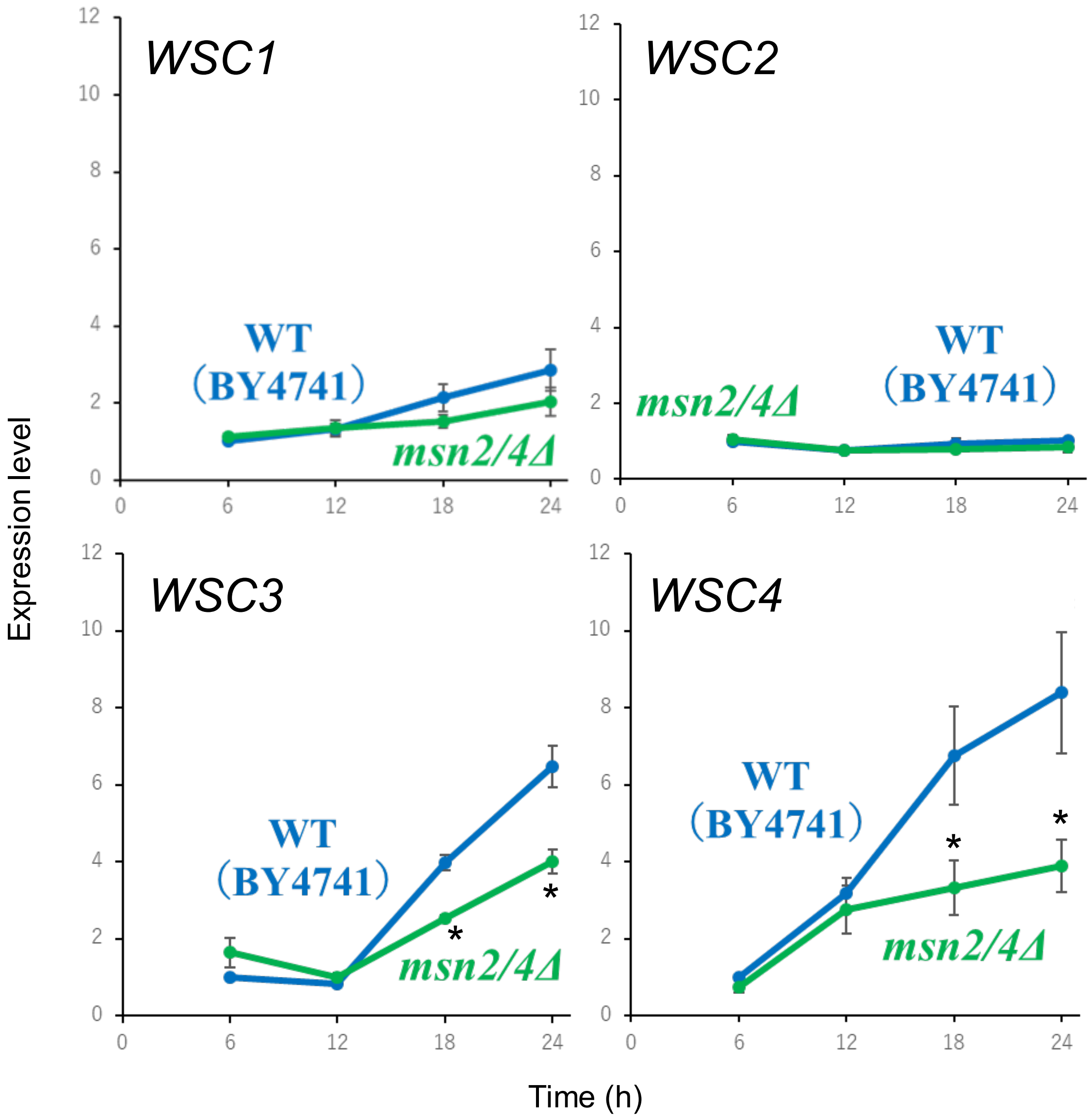

Supplementary Figure 6 Expression of the *WSC3* and *WSC4* genes is induced at the initial stage of alcoholic fermentation in an Msn2/4p-dependent manner. The expression levels were normalized by those of WT/6 h samples. Asterisks denote values that are statistically different from WT as determined by Student's *t* test, $p < 0.05$. The values are the means and standard deviations of three independent experiments.

Supplementary Figure 7 (Watanabe *et al.*)

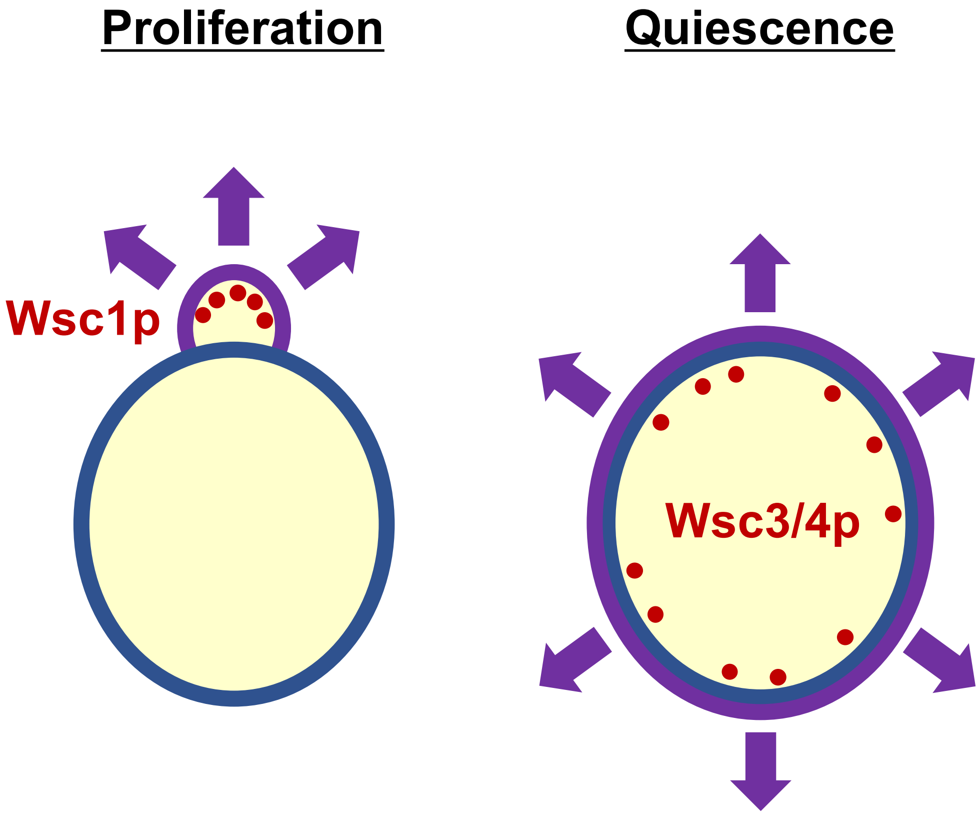

Supplementary Figure 7 Hypothetical model on different roles of Wsc1p and Wsc3/4p as cell wall sensors. At proliferative stage, 1,3-β-glucan synthesis specifically occurs at the budding or growing sites of the cell in a Wsc1p-dependent manner. In contrast, upon the entry into quiescence, cell wall evenly thickens to increase physical strength of the cell via the function of Wsc3/4p.

Supplementary Figure 8 (Watanabe *et al.*)

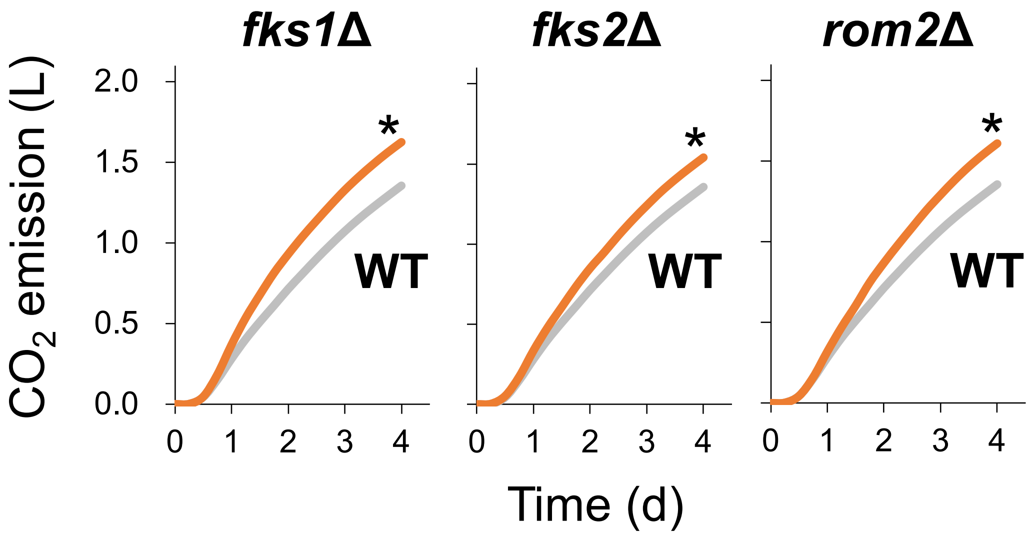

Supplementary Figure 8 Improvement of fermentation capacity by deleting the 1,3-β-glucan synthetic pathway genes. Asterisks denote values that are statistically different from WT as determined by Student's *t* test, $p < 0.05$.

Supplementary Figure 9 (Watanabe *et al.*)

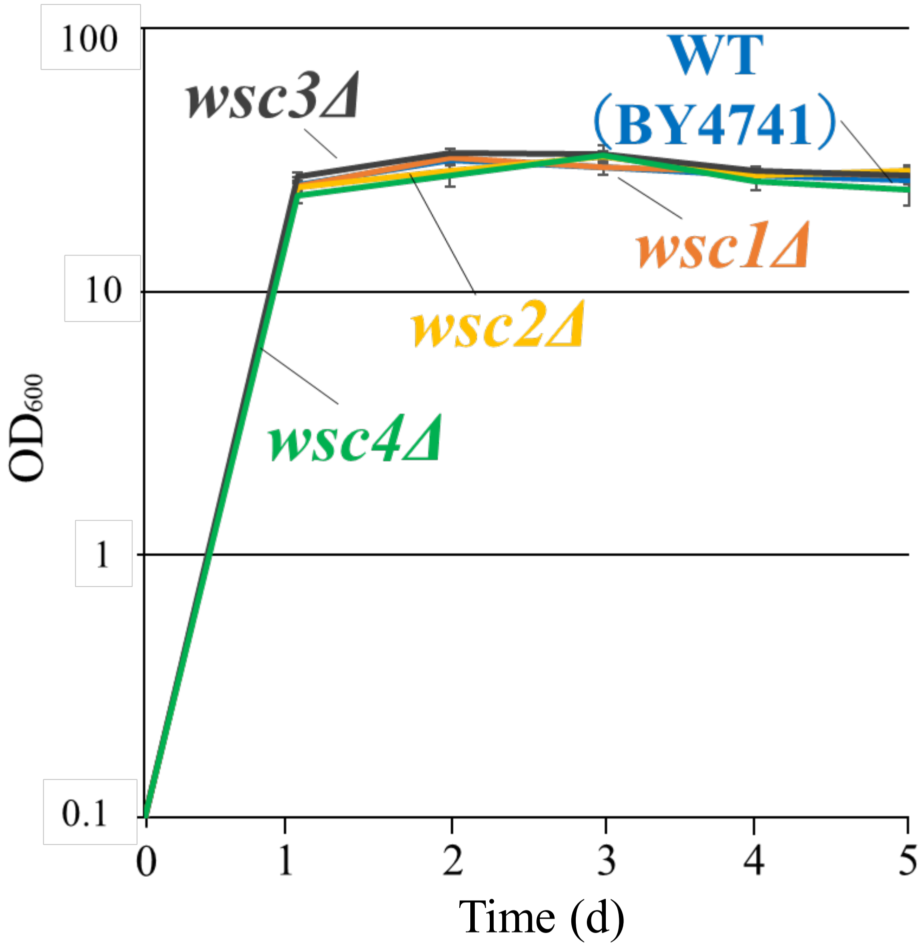

Supplementary Figure 9 None of the *wsc*Δ mutations does not alter yeast growth during alcoholic fermentation. The values are the means and standard deviations of three independent experiments.

Supplementary Figure 10 (Watanabe *et al.*)

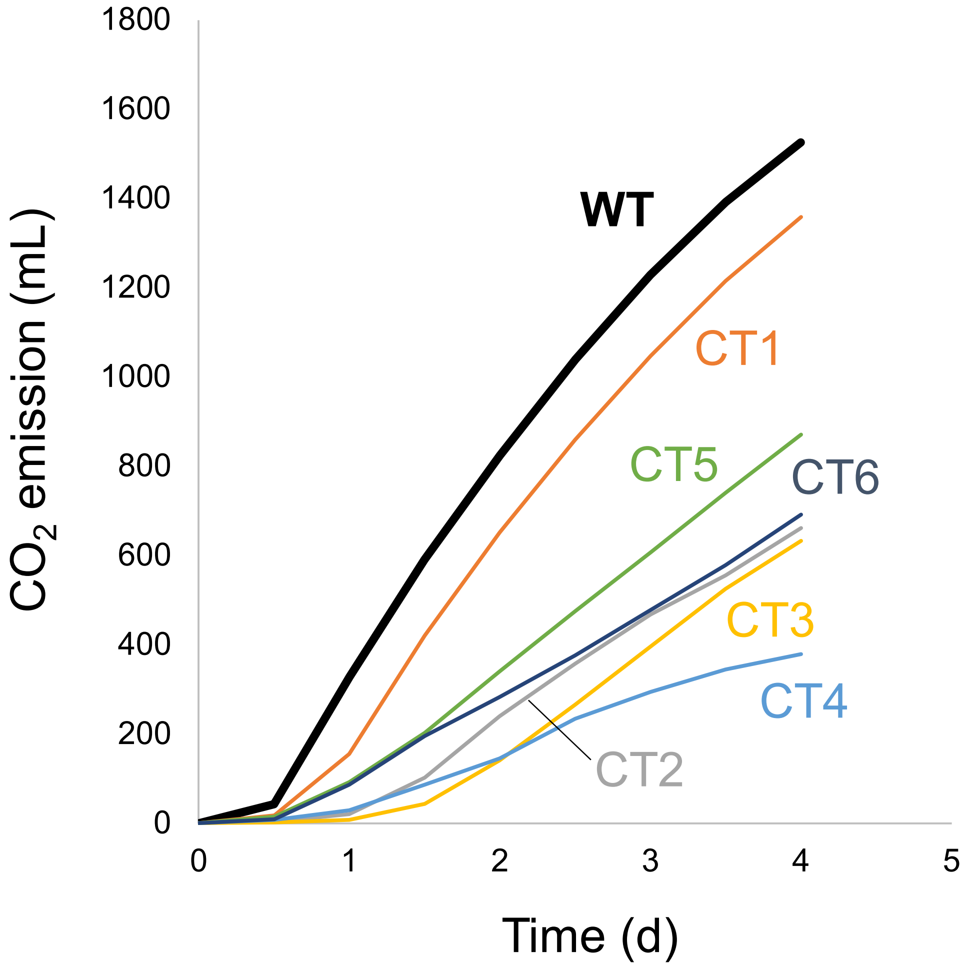

Supplementary Figure 10 Fermentation rates of six randomly isolated caspofungin-toletant (CT) mutants of a laboratory strain X2180. Yeast cells were statically incubated in YPD20 medium without shaking to monitor carbon dioxide emission during alcoholic fermentation.

Supplementary Figure 11 (Watanabe *et al.*)

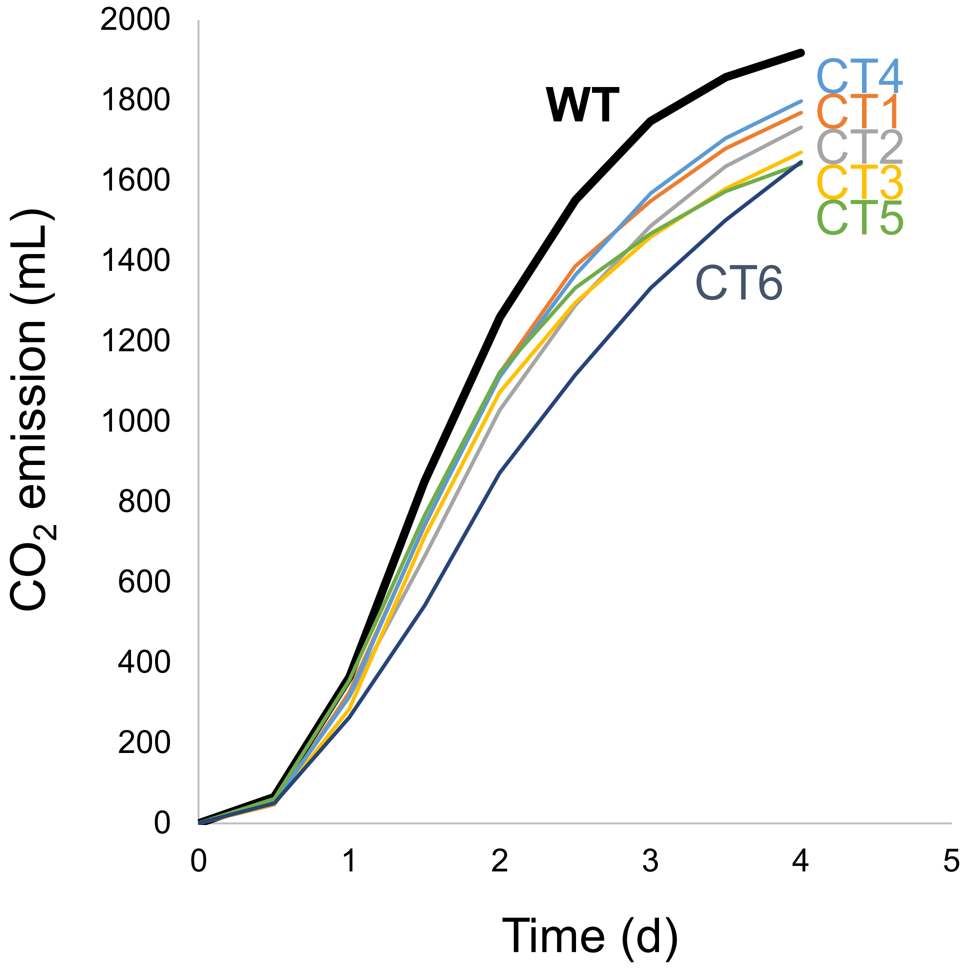

Supplementary Figure 11 Fermentation rates of six randomly isolated caspofungin-toletant (CT) mutants of a sake strain K701. Yeast cells were statically incubated in YPD20 medium without shaking to monitor carbon dioxide emission during alcoholic fermentation. The CT6 mutant corresponds to the caspofungin tolerant mutant, used for sake fermentation test in Figure 2.

Supplementary Figure 12 (Watanabe *et al.*)

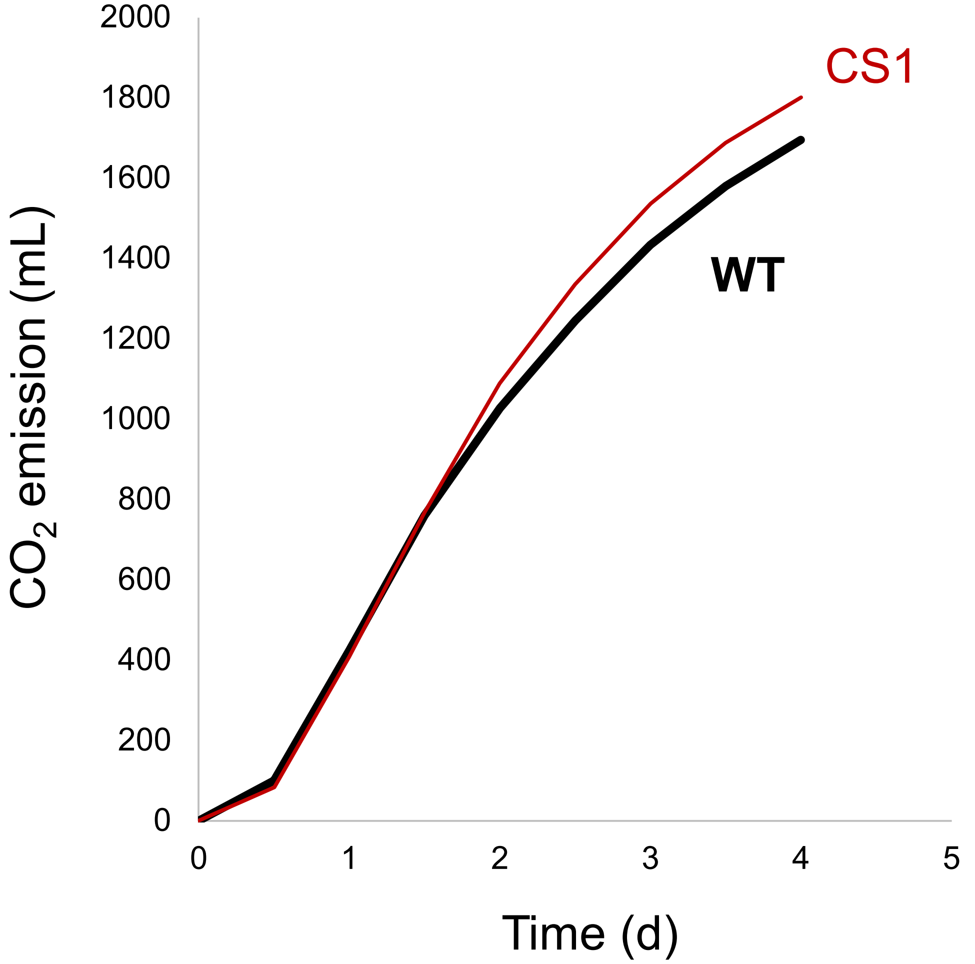

Supplementary Figure 12 Fermentation rate of an isolated caspofungin-sensitive (CS) mutant of a sake strain K701. Yeast cells were statically incubated in YPD20 medium without shaking to monitor carbon dioxide emission during alcoholic fermentation.

Supplementary Table 1 Differentially expressed genes (DEGs) between WT and *cdc55*Δ in the early stage of alcoholic fermentation.

a) Genes with increased expression in *cdc55*Δ

gene_id	a.value	m.value	p.value	q.value	rank	estimat	Genbank_ID	Gbkey	Gene	Product
rna1886	11.46131347	-3.181685958	6.46039E-32	6.91262E-29	6	1	NM_001179952.1	mRNA	*HSP12*	lipid-binding protein HSP12
rna2317	5.691278499	-2.909059644	0.000463928	0.026126453	114	1	NM_001181195.3	mRNA	*GID10*	hypothetical protein
rna2337	10.37089319	-2.503002934	5.78739E-18	5.30786E-15	7	1	NM_001181217.1	mRNA	*CTT1*	catalase T
rna2717	9.802794853	-2.5026993	6.61454E-16	5.30817E-13	8	1	NM_001179217.1	mRNA	*RTC3*	Rtc3p
rna1719	7.951525071	-2.484577245	1.59385E-08	3.32986E-06	30	1	NM_001178987.1	mRNA	*SHC1*	Shc1p
rna6349	10.47964	-2.285509312	1.37185E-15	9.78583E-13	9	1	NM_001184257.1	mRNA	*GPH1*	glycogen phosphorylase
rna3204	10.15553045	-2.202317033	1.06384E-13	6.20897E-11	11	1	NM_001181541.1	mRNA	*PRM10*	pheromone-regulated protein PRM10
rna5039	6.897440057	-2.151457757	0.000290287	0.018093601	103	1	NM_001183032.1	mRNA	*YNL194C*	hypothetical protein
rna3205	10.47906056	-2.140769061	6.88961E-14	4.42313E-11	10	1	NM_001181540.1	mRNA	*YJL107C*	hypothetical protein
rna2117	8.121464952	-2.134186033	5.74819E-07	8.20075E-05	45	1	NM_001180986.1	mRNA	*GPG1*	Gpg1p
rna2510	9.132181637	-2.064971423	2.26641E-09	6.06265E-07	24	1	NM_001181372.1	mRNA	*MPC3*	mitochondrial pyruvate carrier
rna1590	8.88986087	-2.041687955	1.66509E-08	3.34059E-06	32	1	NM_001178826.1	mRNA	*GLC3*	1,4-alpha-glucan branching enzyme
rna2725	7.876245233	-1.991280732	1.24431E-05	0.001210375	66	1	NM_001179226.1	mRNA	*HXT5*	hexose transporter HXT5
rna4040	8.258232976	-1.989368477	1.64335E-06	0.000206869	51	1	NM_001182036.1	mRNA	*GID11*	hypothetical protein
rna3549	9.308751126	-1.97757816	3.65908E-09	9.39652E-07	25	1	NM_001179727.1	mRNA	*KDX1*	putative protein kinase KDX1
rna1030	6.984663128	-1.964759408	0.000761413	0.040011764	122	1	NM_001180378.1	mRNA	*FMP16*	Fmp16p
rna2084	9.9437507	-1.94187679	1.75445E-10	5.2595E-08	21	1	NM_001181021.1	mRNA	*AMS1*	alpha-mannosidase
rna2515	8.162085844	-1.923726086	6.14023E-06	0.000646235	61	1	NM_001181377.3	mRNA	*SOL4*	6-phosphogluconolactonase SOL4
rna4060	8.001439408	-1.894134946	1.90204E-05	0.001719872	71	1	NM_001184560.1	mRNA	*YLR154C-G*	hypothetical protein
rna1970	10.44541083	-1.887500922	4.37909E-11	1.59049E-08	16	1	NM_001180018.3	mRNA	*HXK1*	hexokinase 1
rna5685	8.649938738	-1.791828735	2.72422E-06	0.000323879	54	1	NM_001183604.1	mRNA	*GSP2*	Ran GTPase GSP2
rna2295	8.524133457	-1.790134713	5.14104E-06	0.000550092	60	1	NM_001181172.1	mRNA	*NQM1*	sedoheptulose-7-phosphate:D-glyceraldehyde-3-phosphate transaldolase NQM1
rna5660	12.36618386	-1.77252239	1.6329E-12	8.064E-10	13	1	NM_001183580.3	mRNA	*PNS1*	Pns1p
rna2848	8.145356281	-1.763615088	3.97475E-05	0.003314016	77	1	NM_001179340.1	mRNA	*CRG1*	S-adenosylmethionine-dependent methyltransferase
rna3091	8.446134753	-1.731956507	1.50483E-05	0.001420737	68	1	NM_001179561.3	mRNA	*YPS6*	aspartyl protease
rna2082	8.328458379	-1.672417938	4.88971E-05	0.003923989	80	1	NM_001181023.1	mRNA	*RCK1*	putative serine/threonine protein kinase RCK1
rna4658	10.56134133	-1.65907424	4.31949E-09	1.02708E-06	27	1	NM_001182605.1	mRNA	*PGM2*	phosphoglucomutase PGM2
rna849	9.728777764	-1.650544989	1.48808E-07	2.44961E-05	39	1	NM_001180169.1	mRNA	*TMA17*	Tma17p
rna742	8.716677444	-1.637726574	1.46033E-05	0.001399297	67	1	NM_001180282.1	mRNA	*FMP45*	Fmp45p
rna3147	9.256586036	-1.605213285	2.32772E-06	0.000281962	53	1	NM_001181596.1	mRNA	*YJL163C*	hypothetical protein
rna781	8.277670827	-1.585989272	0.000146883	0.010249854	92	1	NM_001180241.1	mRNA	*INH1*	ATPase inhibitor
rna4761	11.4226238	-1.525938767	5.94872E-09	1.36396E-06	28	1	NM_001182703.1	mRNA	*YMR196W*	hypothetical protein
rna3612	10.53738632	-1.492097575	1.35681E-07	2.2923E-05	38	1	NM_001179662.1	mRNA	*CWP1*	Cwp1p
rna1046	9.249186578	-1.454071363	2.00736E-05	0.001789897	72	1	NM_001180393.1	mRNA	*AFR1*	Afr1p
rna6164	8.207202932	-1.445053483	0.000704309	0.037680553	120	1	NM_001183831.1	mRNA	*IRC15*	Irc15p
rna6063	8.942719071	-1.439118916	6.97082E-05	0.005457645	82	1	NM_001183933.1	mRNA	*DBP1*	putative DEAD-box ATP-dependent RNA helicase DBP1
rna4401	10.99355703	-1.396550764	2.47969E-07	3.88284E-05	41	1	NM_001182491.1	mRNA	*MSC1*	Msc1p
rna829	8.270037449	-1.394010767	0.000918967	0.046823563	126	1	NM_001184325.1	mRNA	*STF1*	ATPase-binding protein
rna4820	11.13605764	-1.375814654	2.68521E-07	4.10454E-05	42	1	NM_001182756.1	mRNA	*GAD1*	glutamate decarboxylase GAD1
rna563	13.15103264	-1.372854771	1.60788E-08	3.32986E-06	31	1	NM_001178685.1	mRNA	*GLK1*	glucokinase
rna3090	10.53903395	-1.369268301	1.29841E-06	0.000166716	50	1	NM_001179560.1	mRNA	*GTT1*	bifunctional glutathione transferase/peroxidase
rna1034	13.31614273	-1.359164195	1.9545E-08	3.80239E-06	33	1	NM_001180382.1	mRNA	*TPS2*	trehalose-phosphatase TPS2
rna1487	11.08110193	-1.346853446	5.31187E-07	7.7505E-05	44	1	NM_001180824.3	mRNA	*EMI2*	putative glucokinase
rna5070	13.29348363	-1.328835062	3.98908E-08	7.53233E-06	34	1	NM_001182998.1	mRNA	*YGP1*	Ygp1p
rna5873	12.02525593	-1.316388218	1.86544E-07	2.99404E-05	40	1	NM_001183794.1	mRNA	*ALD4*	aldehyde dehydrogenase (NADP(+)) ALD4
rna932	8.878314497	-1.310309769	0.0003624	0.021542654	108	1	NM_001180080.1	mRNA	*GPM2*	phosphoglycerate mutase family protein GPM2
rna2903	9.711623178	-1.289035507	4.44411E-05	0.00365784	78	1	NM_001179484.1	mRNA	*OM45*	Om45p
rna4431	12.03851513	-1.253681541	6.63738E-07	9.06638E-05	47	1	NM_001182460.1	mRNA	*TSL1*	trehalose 6-phosphate synthase/phosphatase complex subunit
rna1678	12.88154158	-1.228251535	4.8458E-07	7.23489E-05	43	1	NM_001178953.3	mRNA	*GPP2*	glycerol-1-phosphatase HOR2
rna562	11.19335176	-1.222839279	4.23969E-06	0.000477524	57	1	NM_001178687.1	mRNA	*YCL042W*	hypothetical protein
rna2251	10.18021111	-1.221508993	3.51668E-05	0.002970672	76	1	NM_001181137.1	mRNA	*STF2*	ATPase-stabilizing factor family protein
rna1645	10.99274215	-1.194262735	9.96664E-06	0.000984397	65	1	NM_001178928.1	mRNA	*PHM8*	bifunctional nucleotidase/lysophosphatidic acid phosphatase
rna2205	12.86725716	-1.194170957	9.90534E-07	0.00012978	49	1	NM_001180902.3	mRNA	*PNC1*	nicotinamidase
rna931	13.91016323	-1.186526479	6.59605E-07	9.06638E-05	46	1	NM_001180081.1	mRNA	*GPD1*	glycerol-3-phosphate dehydrogenase (NAD(+)) GPD1
rna224	10.51669303	-1.172914789	3.50787E-05	0.002970672	75	1	NM_001178354.1	mRNA	*UGA2*	succinate-semialdehyde dehydrogenase (NAD(P)(+))
rna2806	9.66415662	-1.119022956	0.000441921	0.025559752	111	1	NM_001179302.1	mRNA	*ATG7*	Atg7p
rna4180	10.89168071	-1.111694539	4.56866E-05	0.00371276	79	1	NM_001182145.1	mRNA	*GSY2*	glycogen (starch) synthase GSY2
rna3603	10.98199756	-1.085270517	5.98486E-05	0.004743554	81	1	NM_001179669.1	mRNA	*APE1*	metalloaminopeptidase APE1
rna2934	10.38798865	-1.081906627	0.000169613	0.011708784	93	1	NM_001179455.1	mRNA	*PFK26*	Pfk26p
rna4733	13.43089064	-1.073647016	7.78917E-06	0.000793753	63	1	NM_001182678.1	mRNA	*DDR48*	DNA damage-responsive protein 48
rna2930	10.90837077	-1.066188588	8.97548E-05	0.006859834	84	1	NM_001179459.1	mRNA	*COX5B*	cytochrome c oxidase subunit Vb
rna2652	11.80397261	-1.065400107	2.88141E-05	0.002534062	73	1	NM_001179160.1	mRNA	*SLT2*	mitogen-activated serine/threonine-protein kinase SLT2
rna4000	10.99269158	-1.063882462	8.19826E-05	0.006341308	83	1	NM_001181996.1	mRNA	*AHP1*	thioredoxin peroxidase AHP1
rna2406	9.925517793	-1.029684368	0.000766581	0.040011764	123	1	NM_001181278.1	mRNA	*GPC1*	glycerophosphocholine acyltransferase
rna3146	10.57691153	-1.027228721	0.000263226	0.016899103	100	1	NM_001181597.2	mRNA	*TPK1*	cAMP-dependent protein kinase catalytic subunit TPK1
rna1037	11.15835712	-1.012578395	0.000142735	0.010249854	89	1	NM_001180385.3	mRNA	*SED1*	Sed1p
rna4193	11.14937159	-1.011560316	0.000146598	0.010249854	91	1	NM_001182157.1	mRNA	*DCS1*	5'-(N(7)-methyl 5'-triphosphoguanosine)-(mRNA) diphosphatase
rna3244	10.10563308	-1.005124382	0.000754388	0.040011764	121	1	NM_001181503.3	mRNA	*YJL070C*	metallo-dependent hydrolase superfamily protein
rna3649	10.5891595	-1.001323554	0.000368101	0.021680817	109	1	NM_001179628.1	mRNA	*MSN4*	stress-responsive transcriptional activator MSN4
rna5117	10.84423223	-0.997918184	0.000267737	0.017018547	101	1	NM_001182953.1	mRNA	*YNL115C*	hypothetical protein
rna2303	11.68358724	-0.993440267	0.000106575	0.008049515	85	1	NM_001181181.1	mRNA	*FMP48*	protein kinase FMP48
rna374	11.93963163	-0.970335082	0.000122091	0.009114202	86	1	NM_001178497.3	mRNA	*ARA1*	D-arabinose 1-dehydrogenase (NAD(P)(+)) ARA1
rna351	12.40358922	-0.939050846	0.000144343	0.010249854	90	1	NM_001178474.1	mRNA	*TPS1*	alpha,alpha-trehalose-phosphate synthase (UDP-forming) TPS1
rna3559	11.30879973	-0.929441478	0.00040799	0.02381179	110	1	NM_001179716.1	mRNA	*MCR1*	cytochrome-b5 reductase
rna3849	12.51383225	-0.904885979	0.00023355	0.015299896	98	1	NM_001181859.1	mRNA	*UBI4*	ubiquitin
rna836	12.17481042	-0.893218897	0.000342066	0.020523986	107	1	NM_001180183.1	mRNA	*YDL124W*	aldo-keto reductase superfamily protein
rna6177	12.98995417	-0.866615232	0.000342007	0.020523986	106	1	NM_001183818.1	mRNA	*LSP1*	lipid-binding protein LSP1
rna3676	13.3085174	-0.795506105	0.000909278	0.046700493	125	1	NM_001179601.3	mRNA	*UGP1*	UTP glucose-1-phosphate uridylyltransferase

b) Genes with decreased expression in *cdc55*Δ

gene_id	a.value	m.value	p.value	q.value	rank	estimat	Genbank_ID	Gbkey	Gene	Product
rna2051	5.678694947	11.17633812	7.08878E-76	4.551E-72	1	1	NM_001181055.1	mRNA	*CDC55*	protein phosphatase 2A regulatory subunit CDC55
rna2860	9.684342801	3.814632776	2.0534E-33	2.63656E-30	5	1	NM_001179346.1	mRNA	*PHO12*	acid phosphatase PHO12
rna2767	6.812439348	3.727864934	4.42984E-11	1.59049E-08	17	1	NM_001179266.1	mRNA	*SPL2*	Spl2p
rna4609	11.58557067	3.641705036	1.41793E-40	3.03436E-37	3	1	NM_001182556.1	mRNA	*FET3*	ferroxidase FET3
rna4406	12.95372874	3.282023437	7.12472E-37	1.14352E-33	4	1	NM_001182486.1	mRNA	*PHO84*	phosphate transporter PHO84
rna1250	6.671623536	3.253802679	6.91617E-08	1.20005E-05	37	1	NM_001180589.1	mRNA	*PHM6*	Phm6p
rna100	8.173363148	2.682740553	1.16658E-10	3.74471E-08	20	1	NM_001178239.1	mRNA	*PHO11*	acid phosphatase PHO11
rna525	6.461079483	2.322200464	0.000587934	0.032261019	117	1	NM_001178644.1	mRNA	*PHO89*	Pho89p
rna2872	6.457619776	2.31528105	0.000621788	0.033829466	118	1	NM_001179517.1	mRNA	*CSS1*	Css1p
rna4026	6.739399279	2.188569083	0.000455304	0.026098679	112	1	NM_001182023.1	mRNA	*TIS11*	Tis11p
rna1795	9.584303972	2.185797299	9.42848E-12	4.32363E-09	14	1	NM_001184429.1	mRNA	*YER159C-A*	gag protein
rna1767	9.6283867	2.148672883	1.55402E-11	6.65121E-09	15	1	NM_001184428.1	mRNA	*YER137C-A*	gag protein
rna5481	9.579726273	2.043189674	2.01142E-10	5.61449E-08	23	1	NM_001183268.1	mRNA	*YOL014W*	hypothetical protein
rna1527	9.915379357	2.015739696	4.45932E-11	1.59049E-08	18	1	NM_001178880.3	mRNA	*SIT1*	siderophore transporter
rna5924	7.06214377	1.94323577	0.000699275	0.037680553	119	1	NM_001184391.1	mRNA	*YPL257W-A*	gag protein
rna322	10.32339926	1.853623362	1.80232E-10	5.2595E-08	22	1	NM_001178441.3	mRNA	*PHO5*	acid phosphatase PHO5
rna5816	10.8673276	1.78831733	7.75725E-11	2.62113E-08	19	1	NM_001183735.1	mRNA	*SFG1*	Sfg1p
rna6162	13.13064147	1.76869729	5.67152E-13	3.03427E-10	12	1	NM_001183833.1	mRNA	*VTC3*	vacuolar transporter chaperone
rna281	7.909338373	1.683065129	0.000236688	0.015348864	99	1	NM_001270739.1	mRNA	*MNC1*	hypothetical protein
rna1775	11.39646831	1.549121925	3.85148E-09	9.51021E-07	26	1	NM_001179035.1	mRNA	*FTR1*	high-affinity iron permease FTR1
rna5329	11.04313924	1.471734984	4.97307E-08	9.12204E-06	35	1	NM_001183411.1	mRNA	*ENB1*	Enb1p
rna630	11.04068598	1.459563535	6.42061E-08	1.14501E-05	36	1	NM_001178735.1	mRNA	*HSP30*	Hsp30p
rna2581	10.28363369	1.390511941	1.87326E-06	0.000231276	52	1	NM_001179108.1	mRNA	*WSC4*	Wsc4p
rna1433	9.846290158	1.331303706	1.70698E-05	0.001588231	69	1	NM_001180769.3	mRNA	*MFA1*	mating pheromone a
rna5885	10.23399975	1.323348872	6.52535E-06	0.000675689	62	1	NM_001183805.3	mRNA	*YOR385W*	hypothetical protein
rna5606	10.39591734	1.319466035	4.53793E-06	0.000502302	58	1	NM_001183526.3	mRNA	*RGS2*	GTPase-activating protein RGS2
rna1983	10.47305943	1.302429032	4.95699E-06	0.000539388	59	1	NM_001181121.1	mRNA	*ZRT1*	high-affinity Zn(2+) transporter ZRT1
rna6163	8.915640189	1.260714898	0.000562856	0.031151141	116	1	NM_001183832.1	mRNA	*CTF19*	Ctf19p
rna2563	10.05557635	1.25485712	2.97256E-05	0.002578895	74	1	NM_001179127.2	mRNA	*ARN2*	siderophore transporter
rna1689	10.54864528	1.21274071	1.7917E-05	0.001643243	70	1	NM_001178963.1	mRNA	*VTC1*	Vtc1p
rna3305	13.23417047	1.1882011	8.83202E-07	0.000118128	48	1	NM_001181446.1	mRNA	*VTC4*	Vtc4p
rna918	9.749670325	1.180732322	0.000173172	0.011827307	94	1	NM_001180096.1	mRNA	*BSC1*	Bsc1p
rna5479	11.73449073	1.138308774	8.7688E-06	0.000879621	64	1	NM_001183270.1	mRNA	*CMK2*	calmodulin-dependent protein kinase CMK2
rna5300	9.28601616	1.124990155	0.000953781	0.048048593	127	1	NM_001183237.1	mRNA	*FRE4*	ferric-chelate reductase
rna3471	10.2109545	1.067177644	0.000289169	0.018093601	102	1	NM_001181805.1	mRNA	*HMS2*	Hms2p
rna6339	10.67994294	1.060145192	0.000141859	0.010249854	88	1	NM_001184254.1	mRNA	*TDA6*	Tda6p
rna2569	10.50987693	1.049066555	0.000219196	0.014507627	97	1	NM_001179120.1	mRNA	*ARN1*	siderophore transporter
rna4891	10.93786989	1.012762487	0.000191973	0.012841372	95	1	NM_001182829.1	mRNA	*DIA1*	Dia1p
rna2776	11.36773964	0.976990835	0.000192021	0.012841372	96	1	NM_001179274.1	mRNA	*DSE2*	Dse2p
rna2501	13.29276934	0.921131615	0.000126681	0.00934819	87	1	NM_001181363.1	mRNA	*YHB1*	flavohemoglobin
rna2500	11.38861984	0.86317549	0.000957978	0.048048593	128	1	NM_001181362.3	mRNA	*PHO81*	Pho81p
rna1745	13.20741988	0.862976324	0.000335284	0.020500203	105	1	NM_001179014.3	mRNA	*DSE1*	Dse1p
rna4895	12.46971522	0.857266871	0.000498996	0.027856972	115	1	NM_001182832.1	mRNA	*FET4*	Fet4p
rna5085	11.80225686	0.84919157	0.000839967	0.043488606	124	1	NM_001182983.1	mRNA	*MFA2*	mating pheromone a

Supplementary Table 2 Top 5 estimated transcription factors responsible for DEGs.

a) Genes with increased expression in *cdc55*Δ

Rank	Transcription Factor	% in user set	% in *S. cerevisiae*	p-value	Target ORF/Genes
1	Gis1p	50.00%	5.52%	0	*FMP45 YDL124W TMA17 GPM2 FMP16 TPS2 GLC3 GPP2 SHC1 HSP12 RCK1 GPG1 STF2 NQM1 FMP48 GID10 CTT1 RTC3 HXT5 CRG1 OM45 COX5B GTT1 YPS6 TPK1 YJL163C APE1 CWP1 GID11 MSC1 PGM2 DDR48 YMR196W GAD1 YNL194C YGP1 LSP1 GPH1 UGA2*
2	Msn4p	87.18%	2.74%	0	*TPS1 ARA1 YCL042W GLK1 FMP45 STF1 YDL124W TMA17 GPD1 GPM2 FMP16 TPS2 EMI2 GLC3 PHM8 GPP2 SHC1 HSP12 HXK1 RCK1 AMS1 GPG1 PNC1 STF2 NQM1 FMP48 GID10 CTT1 MPC3 SOL4 RTC3 HXT5 CRG1 OM45 COX5B PFK26 GTT1 YPS6 TPK1 YJL163C PRM10 YJL107C MCR1 APE1 CWP1 MSN4 UGP1 UBI4 AHP1 GID11 GSY2 DCS1 MSC1 TSL1 PGM2 DDR48 YMR196W GAD1 YNL194C YGP1 PNS1 GSP2 ALD4 DBP1 IRC15 LSP1 GPH1 UGA2*
3	Msn2p	93.59%	2.24%	0	*TPS1 ARA1 YCL042W GLK1 FMP45 STF1 YDL124W TMA17 GPD1 GPM2 FMP16 TPS2 SED1 EMI2 GLC3 PHM8 GPP2 SHC1 HSP12 HXK1 RCK1 AMS1 GPG1 PNC1 STF2 NQM1 FMP48 GID10 CTT1 GPC1 MPC3 SOL4 RTC3 HXT5 CRG1 OM45 COX5B PFK26 GTT1 YPS6 TPK1 YJL163C PRM10 YJL107C KDX1 MCR1 APE1 CWP1 MSN4 UGP1 UBI4 AHP1 GID11 GSY2 DCS1 MSC1 TSL1 PGM2 DDR48 YMR196W GAD1 YNL194C YGP1 YNL115C PNS1 GSP2 ALD4 DBP1 IRC15 LSP1 GPH1 UGA2 YJL070C*
4	Rlm1p	79.49%	8.06%	0	*TPS1 GLK1 FMP45 STF1 YDL124W TMA17 GPD1 GPM2 FMP16 TPS2 SED1 AFR1 GLC3 PHM8 GPP2 HSP12 HXK1 RCK1 AMS1 GPG1 PNC1 STF2 NQM1 FMP48 GID10 CTT1 GPC1 MPC3 SOL4 SLT2 RTC3 HXT5 CRG1 OM45 COX5B PFK26 GTT1 YPS6 PRM10 YJL107C KDX1 MCR1 CWP1 MSN4 UGP1 GID11 GSY2 DCS1 MSC1 TSL1 PGM2 DDR48 YMR196W GAD1 YNL194C YGP1 YNL115C PNS1 ALD4 LSP1 GPH1 UGA2*
5	Fhl1p	69.23%	2.70%	1E-15	*TPS1 ARA1 YCL042W GLK1 FMP45 INH1 STF1 YDL124W TMA17 GPD1 GPM2 FMP16 TPS2 AFR1 EMI2 GLC3 PHM8 GPP2 HSP12 HXK1 AMS1 GPG1 PNC1 STF2 NQM1 GID10 CTT1 MPC3 SOL4 RTC3 HXT5 CRG1 OM45 COX5B YPS6 TPK1 MCR1 APE1 UGP1 UBI4 GID11 GSY2 DCS1 MSC1 TSL1 PGM2 DDR48 GAD1 YNL194C YGP1 GSP2 ALD4 GPH1 UGA2*

b) Genes with decreased expression in *cdc55*Δ

Rank	Transcription Factor	% in user set	% in *S. cerevisiae*	p-value	Target ORF/Genes
1	Hfi1p	59.09%	1.80%	1.031E-09	*PHO5 PHO89 BSC1 PHM6 MFA1 VTC1 DSE1 FTR1 YHB1 WSC4 SPL2 DSE2 PHO12 VTC4 HMS2 PHO84 DIA1 FET4 ENB1 YOL014W YOR385W VTC3 PHO11 YER137C-A YER159C-A YPL257W-A*
2	Hsf1p	61.36%	1.43%	8.40867E-08	*PHO5 PHO89 HSP30 BSC1 PHM6 MFA1 VTC1 DSE1 FTR1 ZRT1 WSC4 SPL2 DSE2 PHO12 VTC4 HMS2 PHO84 DIA1 FET4 ENB1 YOL014W SFG1 VTC3 PHO11 YER137C-A YER159C-A YPL257W-A*
3	Snf2p	63.64%	1.34%	1.71398E-07	*PHO5 PHO89 BSC1 PHM6 MFA1 SIT1 DSE1 FTR1 ZRT1 YHB1 ARN2 SPL2 DSE2 PHO12 CSS1 VTC4 HMS2 PHO84 FET3 DIA1 ENB1 YOL014W YOR385W VTC3 PHO11 YER137C-A YER159C-A YPL257W-A*
4	Spt3p	45.45%	1.62%	1.40885E-06	*PHO5 PHO89 PHM6 FTR1 ZRT1 YHB1 WSC4 SPL2 DSE2 PHO12 CSS1 VTC4 HMS2 PHO84 ENB1 VTC3 PHO11 YER137C-A YER159C-A YPL257W-A*
5	Upc2p	25.00%	2.64%	3.27097E-06	*BSC1 SIT1 FTR1 ARN2 ARN1 WSC4 PHO12 FET3 FRE4 TDA6 PHO11*

Supplementary Table 3 Primers used in qRT-PCR analysis.

Name	Sequence
CTT1-RT-Fw	CGCCGCTCCATACCAGAAT
CTT1-RT-Rv	CGGTGGAAAAACGAACAAGAC
HSP12-RT-Fw	ACATCACTGACAAGGCCGACA
HSP12-RT-Rv	GCGTTATCCTTGCCTTTTTCG
PGM2-RT-Fw	GGTGACTCCGTCGCAATTAT
PGM2-RT-Rv	CGTCGAACAAAGCACAGAAA
UGP1-RT-Fw	ATCGAGCAATTTGGAGATGG
UGP1-RT-Rv	AACCAGCAACAAATCGGAAC
WSC1-RT-Fw	GCGGGAACAAGACAGGATG
WSC1-RT-Rv	AGTTTGCCCTTTGGTGTGCT
WSC2-RT-Fw	TTCTTCTCCTTCCACCACTTCC
WSC2-RT-Rv	GGGGTAGAAGTGACCGTTGC
WSC3-RT-Fw	TTGGTGTTGTGTTTGGCGTAA
WSC3-RT-Rv	ACCGAACGAATAGGGCTGATAA
WSC4-RT-Fw	CACACGCCTTCACCATCTTC
WSC4-RT-Rv	CGTGCTTGGGTCATTTGGT
ACT1-RT-Fw	GGTTGCTGCTTTGGTTATTGA
ACT1-RT-Rv	TTTTGACCCATACCGACCAT
